# Strategies and motives for resistance to persuasion: an integrative framework

**DOI:** 10.3389/fpsyg.2015.01201

**Published:** 2015-08-14

**Authors:** Marieke L. Fransen, Edith G. Smit, Peeter W. J. Verlegh

**Affiliations:** ^1^Amsterdam School of Communication Research, Department of Communication Science, University of Amsterdam, Amsterdam, Netherlands; ^2^Marketing Department, Vrije Universiteit Amsterdam, Amsterdam, Netherlands

**Keywords:** persuasion, resistance, reactance, deception, change

## Abstract

Persuasion is an important element of human communication. But in many situations, we resist rather than embrace persuasive attempts. Resistance to persuasion has been studied in many different disciplines, including communication science, psychology, and marketing. The present paper reviews and connects these diverse literatures, and provides an organizing framework for understanding and studying resistance. Four clusters of resistance strategies are defined (avoidance, contesting, biased processing, and empowerment), and these clusters are related to different motivations for resisting persuasion (threat to freedom, reluctance to change, and concerns of deception). We propose that, while avoidance strategies may be triggered by any of these motivations, contesting strategies are linked primarily to concerns of deception, while empowerment and biased processing strategies are most common when people are reluctant to change.

## Introduction

Persuasion plays a prominent role in daily life. People frequently try to convince others to change their attitudes, opinions, or behavior. Consider a manager asking one of his employees to work extra hours during the weekend, a politician convincing the public to give him their vote, a doctor encouraging his patients to take their medicines, or a television commercial persuading consumers that they need a safe car to take good care of their beloved families. However, achieving such change is not as easy as it may seem. As Miller (1965) explains, “In our daily lives we are struck not by the ease of producing attitude change but by the rarity of it” (p. 121).

Attempts at persuasion often have limited impact. One of the most important reasons might be that people do not *want* to be influenced; they are motivated to resist persuasion (Ringold, 2002). Motivated resistance does not underlie all instances of attenuation of attitudinal or behavioral change. Persuasion attempts may be poorly designed or executed, or their impact may be reduced by interfering influences from other sources. Following Knowles and Linn (2004, p. 3), we therefore differentiate between motivated resistance and outcome resistance, which is simply defined as “the antithesis of persuasion” or the lack of attitude change in response to a persuasion attempt (cf., Sagarin et al., 2002). Motivated resistance acknowledges that people are armed with resistance strategies that may impede even well designed campaigns. More formally, it entails a state in which people aim to reduce attitudinal or behavioral change and maintain their current attitude. In doing so, people oppose, counter, and resist persuasive attempts by adopting strategies such as counter arguing or avoidance. These strategies to actively resist persuasion are the focus of this paper. Our conceptualization of resistance echoes McGuire (1964), who regarded resistance to persuasion as a property of a person that could be enhanced by message or context factors.

Resistance to persuasion has been studied in many research domains, such as social psychology, marketing, health, and political communication. These domains are intrinsically linked to each other but also show many different approaches to the topic of resistance toward persuasion. Due to this rather disconnected nature of previous work on resistance toward persuasion, we emphasize that we do not claim to provide an exhaustive review of the literature. However, we do propose a preliminary framework that organizes available resistance strategies and motivational factors that explain why people resist and when particular resistance strategies are adopted. The purpose of this article is therefore twofold. First, we review and make a first attempt to synthesize existing literature on resistance. This offers an overview of the strategies that people use to resist unwanted persuasion. Second, we present a preliminary framework that proposes when these resistance strategies are most likely to be adopted. This framework (a) offers a guideline for communication practitioners who aim to persuade people toward, for example, healthier behavior and (b) facilitates the development of resistance programs designed to help vulnerable people resist unwanted persuasion.

This article is structured as follows. First, we present an overview of resistance strategies, explaining how people resist persuasion. In doing so, we organize the existing literature into four main types of strategies that people might adopt when exerting resistance: avoidance strategies, contesting strategies, biased processing strategies, and empowerment strategies. Next, we argue that the type of resistance strategy people adopt depends on the motives they have for resisting the message, namely, threat to freedom, reluctance to change, and concerns about deception. These three motives for people to resist persuasion are introduced and discussed separately in conjunction with message and personality factors that are likely to affect them. Finally, we present a preliminary framework in which the use of the different resistance strategies is predicted by the different resistance motives. This results in a set of propositions describing the relationships between resistance strategies and underlying motives.

## How People Resist Persuasion

This section reviews the different strategies that individuals apply to resist persuasion. We group the strategies into four clusters. The first cluster consists of *avoidance strategies*. These are the most passive strategies, and involve the mere avoidance of persuasion attempts. The second cluster consists of *contesting strategies*. This includes the active challenging of the message, the source, or persuasion tactics used. The third cluster consists of *biased processing strategies*, which involves strategies by which recipients selectively process or understand the message in such way that it favors their original attitudes or behavior. The fourth cluster, *empowerment strategies*, consists of strategies where individuals assert their own, existing views instead of challenging the persuasive communication. Below, we define and discuss these strategies.

### Avoidance Strategies

Avoidance is perhaps the most straightforward means of protecting oneself from the impact of persuasive messages. Avoidance behavior has primarily been studied in the context of marketing communications, where researchers have studied the factors that cause individuals to switch channels (zapping), fast forward commercials in recorded programs (zipping), switch off their television, or leave the room to avoid commercial messages (Brodin, 2007). For example, Woltman et al. (2003) demonstrate in their research that television viewers more often avoid informational messages as opposed to emotional and entertaining messages. Avoidance is not limited to television advertising. Speck and Elliott (1997) discuss avoidance behaviors in several media, including print and radio advertising. They distinguish between *physical avoidance*, whereby people leave the room or avoid the advertising section in a newspaper; *mechanical avoidance* like zapping and zipping; and *cognitive avoidance*, i.e., “ignoring” or “not paying attention to” commercial messages. Dreze and Hussherr (2003, p. 8) describe avoidance of online media. Studying audience eye movements, these authors found that “surfers actually avoid looking at banner ads during their online activities,” also referred to as banner-blindness (Resnick and Albert, 2014). Finally, Kirmani and Campbell (2004) have described physical avoidance in an interpersonal context, and found evidence for a so-called “forestall strategy,” in which shoppers physically avoid salespersons, for example by crossing the street or avoiding sections where a sales representative walks around.

Researchers in political and health communication have also studied avoidance, in the form of “selective exposure” or “selective avoidance.” This is the tendency to avoid media programming or titles likely to contain messages contradicting one’s own beliefs (e.g., Freedman and Sears, 1965; Knobloch-Westerwick and Meng, 2009). Festinger’s (1957) cognitive dissonance theory regards this behavior as a strategy for decreasing the dissonance that people experience due to inconsistencies. This experienced dissonance can be reduced by avoiding inconsistent information or searching for new consistent information. For example, Brock and Balloun (1967) showed that people who smoke paid more attention to a message stating that smoking is not detrimental to their health than to a message stating that smoking is a serious health risk. The opposite pattern was found for people who do *not* smoke. The link between cognitive dissonance and selective exposure has been examined in many studies. Meta-analyses of this work (e.g., Freedman and Sears, 1965; Frey, 1986; D’Alessio and Allen, 2007; Hart et al., 2009) emphasize the importance of considering moderating variables for this effect. One of the most important moderaters is attitude strength or extremity. Consistent with the notion of cognitive dissonance, selective exposure behavior seems more likely for individuals with a stronger opinion. For example, Brannon et al. (2007) demonstrated that participants preferred reading articles with titles that were consistent with their own attitudes, and this tendency increased with the extremity of their attitudes. Knobloch-Westerwick and Meng (2009) obtained similar findings when tracking reading behavior in an online environment. In addition to attitude strength, a wide range of message and audience characteristics moderate the selective exposure effect (Smith et al., 2008).

### Contesting Strategies

Instead of avoiding the message, individuals may actively contest (a) the content of the message, (b) the source of the message, or (c) the persuasive strategies used in the message. Below we discuss these three forms of contestation.

#### Contesting the Content

A frequently used resistance strategy is to counter argue the message (e.g., Wright, 1975; Zuwerink Jacks and Cameron, 2003). We refer to this behavior as “contesting the content” to emphasize that this strategy is closely related to source derogation (contesting the source of a message) and to defensive responses studied in consumer research (contesting the persuasive strategy). Contesting the content of a message is a thought process that decreases agreement with a counter attitudinal message. It is often conceptualized as a mediating variable between a persuasive message and outcomes such as attitudes and behavior (Festinger and Maccoby, 1964; Silvia, 2006). When contesting the content of a message, people reflect on the arguments in the message and subsequently use counterarguments to refute it. Counterarguments are activated when incoming information is compared to existing beliefs and discrepancies are noted (Wright, 1973). Counter arguing can be encouraged by forewarning (Wood and Quinn, 2003), i.e., the (upfront) disclosure of the persuasive intent and/or content of a message. The effectiveness of forewarning increases when a greater time delay occurs between the warning and the message, because this gives them the opportunity to generate counterarguments (e.g., Chen et al., 1992). Consistent with this finding, recent research demonstrated that counter arguing is less likely for narratives because the persuasive intentions are less clear for such communications. However, counter arguing may be triggered when the narrative is combined with elements revealing the persuasive intent of the message (Moyer-Gusé and Nabi, 2011; Niederdeppe et al., 2012).

#### Contesting the Source

In addition to contesting content, individuals may contest the source of a message. This behavior has been referred to as source derogation, and involves dismissing the credibility of sources or questioning their expertise or trustworthiness (Abelson and Miller, 1967; Zuwerink Jacks and Cameron, 2003). In earlier research on persuasion, source derogation was perceived as a communication strategy that could be used to reduce or counter the effect of persuasion attempts (e.g., Anderson, 1967). In later research, Wright (1973, 1975) demonstrated that source derogation may be used as a cognitive response to persuasion attempts. Wright regards source derogation as a low-effort alternative to counter arguing because it requires processing of one single cue—the source of the message. Source derogation also underlies the observation that information from commercial sources (e.g., advertising) is viewed as less trustworthy than information from non-commercial sources (e.g., other consumers—Batinic and Appel, 2013). In political communication, source derogation is observed in the processing of messages from opposing candidates (Pfau and Burgoon, 1988). Related to source derogation is the idea of defensive stereotyping. Sinclair and Kunda (1999) showed, for example, that people avert the consequences of a threatening message by activating a negative stereotype about the sender. This way the credibility of both the sender and the message reduces.

#### Contesting the Strategies Used

Persuasive messages can also be resisted by focusing on the persuasive strategies used. The Persuasion Knowledge Model (Friestad and Wright, 1994) proposes that people develop theories and beliefs about how persuasion agents try to influence them. For example, many people know that advertisers use babies, puppies, or beautiful models to appeal to emotions. Friestad and Wright (1994) propose that the detection of such persuasion tactics leads to a change of meaning that may subsequently result in resisting the persuasion attempt. Darke and Ritchie (2007) argued that people may even generalize these negative responses from one instance to the other, thereby providing a possible foundation for defensive stereotyping responses (e.g., “all advertising is untruthful”). More recent research revealed that the use of persuasion knowledge as a resistance strategy may also be automatic and unconscious (Laran et al., 2011).

Persuasion knowledge has been found to develop over time, with age and exposure to marketing messages (Wright et al., 2005), although several studies have indicated that even young children possess elementary knowledge of the persuasive tactics used by marketers, which may be accompanied by a corrective (negative) response to persuasion attempts (Buijzen et al., 2010).

### Biased Processing Strategies

To resist persuasive messages people can also engage in biased processing such that a message fits their attitudes and behavior or reduces relevance. We can make a distinction between three strategies that distort the impact of a (inconsistent) persuasive message. The first two strategies, *weighting attributes* and *reducing impact* involve the distortion of information that is inconsistent with a particular attitude or behavior. The final strategy, *optimism bias*, is related to dismissing the relevance of a message.

#### Weighting Attributes

Ahluwalia (2000) showed that people may engage in biased message processing to resist persuasion such that more weight is attached to information that is consistent with one’s attitudes and less weight is attached to inconsistent information. Ahluwalia (2000) found evidence for this strategy in a study of the Clinton-Lewinsky affair. She found that people who were strongly committed to Clinton shifted the importance that they attached to individual traits of politicians. When pro-Clinton voters heard about the affair, they responded by attaching less weight to traits such as honesty and morality, which were jeopardized by the affair, and more weight to unrelated traits like intelligence and strong leadership. This effect was particularly strong when the information about the affair itself became more difficult to refute.

#### Reducing Impact

The impact of information that is inconsistent with one’s current attitudes can also be distorted by actively avoiding a “spillover” or “halo” effect, and isolating judgments of the “focal” attribute from one’s other judgments. Ahluwalia (2000) found that people who are motivated to resist negative information do not display spill-over or halo-effects in their responses to negative information about one particular aspect of an object. This allowed them to minimize the impact of the negative information on their overall evaluation of the object. Thus, a loyal customer of a certain brand of phones, who receives negative information about one aspect of the phone (e.g., signal reception) will only adjust their opinion of this single aspect. For less loyal customers, such information will lead to a spillover or halo effect, so that opinions about other aspects of the phone (e.g., design or durability) will also be affected.

#### Optimism Bias

Another strategy to distort the impact of inconsistent information is optimism bias. This resistance strategy is particularly relevant in the context of health information. It is suggested that message recipients have the tendency to believe that negative things are less likely to happen to them than to others (Weinstein, 1987; Sharot et al., 2011; Shepperd et al., 2013). As a result they tend to downplay the risks or exaggerate the perception of their own ability to control the situation (Chambers and Windschitl, 2004). When a message makes, for example, smokers aware of the detrimental effect of this unhealthy behavior they construe all kinds of reasons why these threats do not apply to them personally and why they are less at risk than others. They might, for example, respond with “While smoking may cause lung cancer, I do not think this risk is very high for me because it does not run in my family.”

### Empowerment Strategies

Empowerment strategies involve empowering or strengthening the self or one’s existing attitudes to reduce one’s vulnerability to external influence attempts. When using these strategies, people search to confirm their confidence in existing beliefs or themselves. Within this category three different strategies can be distinguished. The first two, *attitude bolstering* and *social validation*, aim to reinforce a particular existing attitude. The third empowerment strategy, *self-assertion*, aims to increase one’s general self-confidence. This strategy strengthens self-confidence, and not one particular attitude.

#### Attitude Bolstering

Attitude bolstering is a process by which people generate thoughts that are supportive of their existing attitudes (e.g., Abelson, 1959; Lydon et al., 1988). Upon exposure to messages, recipients reconsider the reasons for their current attitudes and behavior. They do *not* refute or challenge the arguments that are presented in the message For example, a person in favor of the right to abortion can resist a pro-life message by actively thinking about arguments that support the right to abortion rather than countering the arguments in the pro-life message. Recently, Xu and Wyer (2012) demonstrated that it is possible to induce a “bolstering mindset” and that the process of generating affirmative thoughts about one subject may trigger attitude bolstering about other topics.

#### Social Validation

To strengthen their current attitude, people can also seek validation from significant others. Zuwerink Jacks and Cameron (2003) found that people who are presented with a persuasive message that is incongruent with their existing attitude think of others who share their existing beliefs. This confirms their current attitude or behavior and makes them less susceptible to persuasion. Axsom et al. (1987) found that people use responses of their audience as a heuristic cue for the accuracy of their own ideas. In their study, participants were presented with manipulated positive or negative audience feedback to a message. The results indicated that enthusiastic (positive) feedback enhanced the impact of the message.

#### Self Assertions

In their research on resistance strategies, Zuwerink Jacks and Cameron (2003) observed that people may resist persuasion by asserting the self. People who apply this strategy remind themselves that nothing can change their attitudes or behavior because they are confident about them. This phenomenon occurs for two reasons. First, people with high self-esteem are particularly confident about their own opinions and thus less likely to change their attitudes and behavior upon exposure to a persuasive message. Second, sociometer theory (Leary and Baumeister, 2000) argues that persuasion typically occurs because people desire to behave appropriately and therefore avoid disapproval by conforming to the message. People with high self-esteem feel less social pressure to conform because they feel valued and accepted, which reduces their motivation to behave in a socially appropriate manner (Moreland and Levine, 1989).

## Why People Experience Resistance Toward Persuasion

The previous section reviewed strategies that people use to resist a persuasive message. We propose that the type of strategy adopted depends on each person’s specific motive for resisting the message. In this section, we discuss three motives for resistance: threats to freedom, reluctance to change, and concern about deception. These motives derive from various research domains and will be applied to the field of persuasive communication to elucidate why people are motivated to resist a persuasive attempt. In addition, we discuss factors related to the activation of each resistance motive.

### Threats to Freedom

The theory of psychological reactance is one of the best-known frameworks for understanding why people resist persuasion (for reviews, see Burgoon et al., 2002; Rains, 2013). Reactance theory assumes that human beings have an innate desire for autonomy and independence and experience psychological reactance when they sense that their freedom is threatened or eliminated. When people feel that their freedom is threatened, they are motivated to maintain and restore the threatened opinion or behavior (Brehm and Brehm, 1981). Hence, reactance is regarded as the motivational state of a person whose freedom is threatened.

In the context of persuasion, people can feel threatened in their freedom to (a) exhibit particular attitudes and behavior, (b) change their attitudes and behavior, and (c) avoid committing to any position or behavior (Worchel and Brehm, 1970; Brehm and Brehm, 1981). Even when a message is not contrary to existing beliefs or behavior or when the message is in the receiver’s best interest, persuasive attempts are often perceived as an external threat to freedom. This perception of threat could eventually result in so-called “boomerang-effects” in which people distance themselves from the advocated message and are motivated to engage in less (more) of the encouraged (discouraged) behavior. This phenomenon explains why persuasive attempts not only may be ineffective but also may lead to the opposite of the desired results, such as an increase in unhealthy behavior or a decrease in sales (Clee and Wicklund, 1980; Ringold, 2002).

Dillard and Shen (2005) proposed defining reactance at the level of observable affective and cognitive responses. Their research suggests that reactance is best described by an intertwined model in which an affective anger response and a cognitive response of counter arguing are intertwined. This view was confirmed in subsequent experimental studies, as revealed by a recent meta-analysis of 20 different reactance studies (Rains, 2013).

#### Factors Affecting Threats to Freedom

Although psychological reactance was initially perceived as situation specific, Brehm and Brehm (1981) recognized that people vary in the extent to which they experience reactance. Quick et al. (2011, p. 663) describe trait reactant individuals as “…likely to experience state reactance due to their strong need for independence and autonomy, confrontational and rebellious behavior, and a tendency to resist authority in general.” Trait reactance is commonly measured with the Hong Psychological Reactance Scale (Hong and Faedda, 1996), which contains items such as “regulations trigger a sense of resistance in me” and “I become frustrated when I am unable to make free and independent decisions.” People with high psychological reactance will more often be motivated by a threat to freedom than people who score low on this scale.

Several studies revealed that younger people exhibit more reactance than older people (Hong et al., 1994). Older people regard fewer situations as threatening their freedom because they have learned how to cope with the related emotions. In addition, Brehm and Brehm (1981) argued that older people are better at valuing the importance of freedom and are more motivated to exert a freedom than younger people.

In addition to trait reactance and age, several message factors have been found to affect the experience of threat to freedom. In general, threats to freedom are likely to be triggered by any or all message factors that seem to impose a certain behavior or opinion upon the audience. Research on language use suggests that the use of intense, forceful or dogmatic language, and particularly that which threatens choice, in a persuasive message triggers perceived threats to freedom that may subsequently result in boomerang effects (Worchel and Brehm, 1970; Buller et al., 2000; Dillard and Shen, 2005). Examples of language that threatens choice include phrases such as “No other conclusion makes any sense” and “There is a problem, and you have to be part of the solution” (Dillard and Shen, 2005; see also Quick and Stephenson, 2007). Kronrod et al. (2012) have used the term “assertive language” to refer to messages that use the imperative form or other wording that leaves no option for refusal (i.e., “you must…”). In their study of messages about environmental issues, these authors found that such language may reduce compliance from individuals who attach little importance to the topic (see also Baek et al., 2015). Moreover, guilt appeals have also been found to induce feelings of anger, which is an essential element of reactance. For example, Englis (1990) found that people who were exposed to a guilt commercial reported lower levels of happiness and higher levels of anger, scorn, and disgust.

Threats to freedom may be prevented by elements of communication that emphasize freedom of choice. In terms of language use, this effect may be achieved by using politeness strategies, such as indirect requests, or by providing suggestions, examples, or hints rather than direct requests (Brown, 1987). Beyond language factors, Shen (2010) has demonstrated that empathy-inducing messages (i.e., using language or visual elements that foster perspective-taking and emotional responses) reduce the extent to which an audience experiences threats to freedom. More broadly, Moyer-Gusé (2008) proposed that entertainment persuasion, which uses narrative communication and induces identification with the main character decreases the extent to which people experience threats to freedom. Interestingly, a recent study by Moyer-Gusé et al. (2012) indicates that these effects may be undone if an explicit appeal is “tagged on” to the narrative message.

### Reluctance to Change

Changing people’s attitudes and behavior is often a difficult process because people are naturally motivated to retain their existing beliefs and behavior. Change involves going from the known to the unknown (Steinburg, 1992) and implies a loss of control over one’s situation, which has been identified as a primary cause for resistance (Conner, 1992). A reluctance to change may be caused by an unwillingness to change, but also by a desire to stay the same. While these two forms seem similar at first sight, the former is related closely to a general idea of rigidity, while the latter is more specific, and may occur primarily for beliefs that are important, and perhaps even central to one’s self. We will elaborate upon this distinction in our discussion of the factors that drive reluctance to change.

A persuasive attempt may also induce consistency concerns (Petty et al., 2004), i.e., a fear that changing a behavior or opinion will lead to inconsistencies with prior beliefs or behaviors. People are unwilling toward the possibility that persuasive information may challenge an important belief. This may go beyond the general notion of avoiding cognitive dissonance (Festinger, 1957). Reasons that may make people reluctant toward change include (a) the desire to not lose something of value, (b) believing that the proposed change does not make sense, (c) perceiving greater risks than benefits, and (d) being satisfied with the current situation (Hultman, 1995; Kotter and Schlesinger, 2008).

#### Factors Affecting Reluctance to Change

Several psychological factors are correlated with individuals’ generalized reluctance to change their attitudes and behaviors. Dogmatism has been related to resistance to change in several studies (e.g., Lau and Woodman, 1995). Dogmatic people are characterized by closed-mindedness and cognitive rigidity. They are often averse to change because they find it difficult to adjust to a new situation. Similarly, research on cultural values (cf., Gudykunst, 1997) suggests that reluctance to change is related to basic value dimensions, such as uncertainty avoidance (Hofstede, 1980). Constructs related to cognitive flexibility and openness are the opposite of closed-mindedness and uncertainty avoidance. Research on organizational behavior has indicated that people with high resilience or flexibility are less likely to experience stress as a result of changes and are therefore less resistant to organizational change (Wanberg and Banas, 2000).

Other research has focused on the factors that enhance people’s reluctance to change specific attitudes and beliefs. Reluctance to change may be greater for attitudes and beliefs that are more important to one. This not only refers to opinions that are based on a more careful elaboration of available arguments but also—and perhaps even more strongly—to beliefs that are tied to one’s self-view, i.e., self-protection motivation (Johnson and Eagly, 1989; Eagly and Chaiken, 1993; Sherman and Cohen, 2002).

### Concerns of Deception

A third motive that might explain why people experience resistance toward persuasion is concerns of deception. People do not like to be fooled. People are keen on regarding their belief system as correct and truthful and are more defensive of their attitudes when they believe these are correct. The desire to hold accurate attitudes and opinions is an important motive when processing information (Petty and Cacioppo, 1979; Chaiken, 1980; Petty et al., 2004). As a result of this desire, people often scrutinize information by searching for supporting information and avoiding conflicting information (Lundgren and Prislin, 1998).

#### Factors Affecting Concerns of Deception

One factor that might increase concerns of deception is persuasion knowledge (Friestad and Wright, 1994). Persuasion knowledge includes information on tactics that are used in persuasive situations, how these tactics might influence attitudes and behavior, which tactics are effective, and the sender’s motives. When persuasion knowledge is activated, it often elicits suspicion about the sender’s motives, skepticism toward message arguments, and perceptions of manipulative or deceptive intent. Therefore, we expect a positive relationship between persuasion knowledge and concerns of deception.

The extent to which people have had negative experiences with persuasive attempts is also expected to be related to concerns of deception. Research has indicated that exposure to deceptive advertising makes people skeptical, even toward unrelated advertisements from other sources (Darke and Ritchie, 2007). Hence, when people are deceived once, they develop negative beliefs about communicators in general, undermining the effectiveness of further persuasive communication (Pollay, 1986). In other words, people who have negative experiences with persuasive attempts are more likely to experience concerns of deception, motivating them to resist persuasion.

Skepticism can be described as a tendency to disbelieve. In a persuasive context, one may be skeptical of the literal truth of message claims, the motives of the sender, the value of the information, the appropriateness of the message for a specific audience (e.g., children) or specific products (e.g., alcohol; Obermiller and Spangenberg, 1998). A positive relationship between skepticism and concerns of deception is therefore expected.

Several message characteristics may trigger concerns of deception. For example, advertisements that use certain types of attention-getting tactics, such as delayed sponsor identification, disclosures, a borrowed interest appeal, or negative or incomplete comparisons, increase perceptions of the firm’s manipulative intent, which may result in less favorable brand evaluations (Jain and Posavac, 2004). Moreover, persuasive attempts that push people into choices that might benefit the communicator rather than the recipient may result in the experience of deception (Koslow, 2000). The suspicion of ulterior motives may affect information processing and impression formation (e.g., Friestad and Wright, 1994). When people become aware of ulterior motives, concerns of deception will increase.

## The Strategies and Motives for Resistance to Persuasion (SMRP) Framework

**FIGURE 1:**
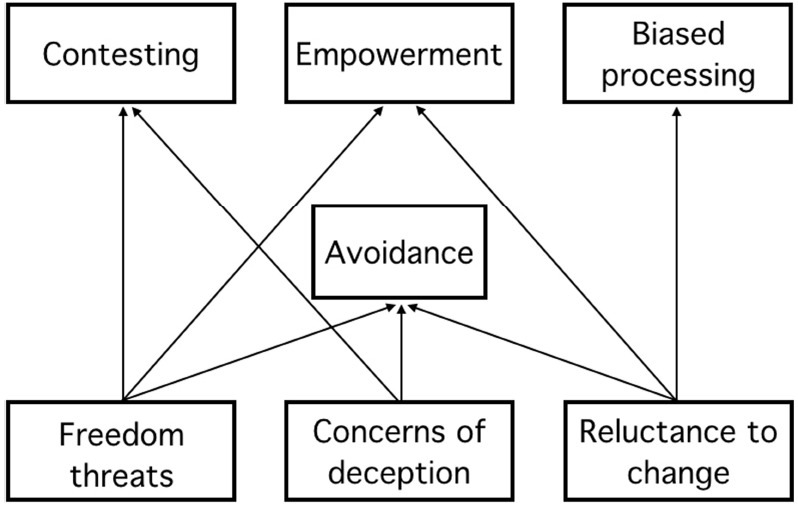
**The SMRP Framework, depicting how resistance motives and resistance strategies are related**.

Having established the motives for resistance, we will discuss how these motives might be related to the use of the different types of resistance strategies (i.e., avoidance strategies, contesting strategies, biased processing strategies, and empowerment strategies) that were presented in the first section of this paper. We establish a general preliminary framework predicting the use of the described resistance strategies by the three different resistance motives. This framework leads to a set of six propositions that define plausible relationships between the underlying motives for resistance and the type of resistance strategy (see Figure 1). Note that many previous studies in different fields have focused on resistance motives and resistance strategies. However, to the best of our knowledge no research empirically tested relationships between different resistance motives and resistance strategies. Previous work either focused on one motive resulting in different resistance strategies or on different motives for one particular resistance strategy. Moreover, we only found one study that examined the use of different resistance strategies by focusing on the likelihood that particular resistance strategies are adopted in a given persuasive situation (Zuwerink Jacks and Cameron, 2003). Our framework should therefore be regarded as a first attempt at organizing the disparate literatures on resistance to persuasion. By no means we claim that the set of propositions is exhaustive and that no additional relationships between specific motivations and specific resistance strategies can be expected. The aim of the framework is to provide a general overview of how resistance motivations and resistance strategies might be related to inspire and guide future research in this domain. In describing the framework, we first explain the use of avoidance strategies and then discuss which strategies each resistance motive is likely to induce. We illustrate these possible relationships by providing examples from the literature that support our hypothesizing.

Avoidance strategies are different from the other types of strategies because they re adopted *before* actual exposure to the persuasion attempt, as opposed to contesting, biased processing and empowerment strategies, which are employed *during* or *after* the attempt. We propose that avoidance strategies may occur with each of the different resistance motives (i.e., threat to freedom, reluctance to change, and concern of deception). Avoidance strategies are particularly adopted when people *anticipate* an unwanted persuasion attempt, whereas the other strategies are used to *cope* with the actual experience of the persuasion attempt, at which point it is too late to adopt avoidance strategies.

Previous literature provides initial evidence for the idea that the three defined resistance motives are related to avoidance strategies. Support for the relationship between reluctance to change and avoidance strategies can be found for example in research demonstrating that people who defend a self-expressive attitude or a core value selectively ignore any information that may threaten this attitude or value (Chaiken et al., 1996). More generally, Sweeney et al. (2010) argue that people avoid information because (inconsistent) information often demands a change in beliefs or an undesired action. A meta-analysis by Hart et al. (2009) demonstrated that selective exposure and avoidance is guided by defense and accuracy motivations. Defense motivation is defined as the desire to defend one’s existing attitudes, beliefs, and behavior because one wants to feel validated and remain consistent beliefs and behaviors (cf. reluctance to change).

Accuracy motivation is related to the motive of concerns of deception, and defined as the desire to form accurate beliefs and attitudes. Both accuracy and defense motivations have been found to initiate selective exposure processes although these relationships depend on various moderators such as relevance, information quality, attitude strength, and attitudinal ambivalence (Sawicki et al., 2013). Research in advertising has also shown that people who rate advertising as deceptive are more inclined to avoid the message (Speck and Elliott, 1997).

Other work in the advertising domain (Edwards et al., 2002) demonstrated that forced exposure to pop-up advertisements leads to a perceived threat to freedom (operationalized as advertisement intrusiveness) and subsequently to advertisement avoidance. In a broader sense, this is reflected in the earlier cited work by Sweeney et al. (2010), who propose that—next to reluctance to change—people may be motivated to avoid inconsistent information because it requires emotion regulation, which plays an important role in coping with threats to freedom.

In sum, in the literature we found support for our notion that avoidance strategies are related to the three defined resistance motives. However, to use the avoidance strategies, people should be aware of the upcoming persuasive event so that they can avoid the activation of the resistance motives.

Proposition 1: Avoidance strategies are likely to be adopted upon the **anticipated** experience of threats to freedom, unwanted requests for change, or the possibility of deception.

It is often not possible to avoid a persuasive message, because such messages are omnipresent in our contemporary environment. In many situations, avoidance strategies are therefore not sufficient, so that contesting, biased processing, and empowerment strategies come into play. We discuss below how the underlying motives are related to these three types of strategies. First, we discuss the relationship between reluctance to change on the one hand, and empowerment and biased processing strategies on the other. Second, we explain how concerns of deception predict the use of contesting strategies, and finally, we describe how threats to freedom are related to both contesting and empowerment strategies.

### Reluctance to Change

We propose that people who are reluctant to change are especially likely to use *empowerment* strategies because these strategies involve resisting persuasive messages by reinforcing either the self (i.e., assertions of confidence) or the particular attitude or belief that is challenged (i.e., attitude bolstering, social validation). Alternatively, they may employ *biased processing* strategies because these focus on processing information in such way that it aligns with existing attitudes and behavior.

The use of empowerment strategies in conditions where people are reluctant to change is illustrated by several examples. In a classic study, Sherman and Gorkin (1980) found that attitude bolstering is more likely to occur when persuasive messages are targeting on attitudes that are more central to the self. From the literature on social influence, we know that social validation is most effective when people feel uncertain about the situation or their attitudes (Cialdini, 2001). Compton and Pfau (2009) postulate that people use “talk as reassurance”: when encountering threatening information, people talk about this information in order to reaffirm their current behaviors and beliefs. This idea was confirmed by Ivanov et al. (2012), who found that the effectiveness of inoculation messages could be increased by allowing people to engage in post-inoculation talk in which people can then validate their attitudes.

Reluctance to change may also induce biased processing strategies including weighting information and reducing impact because people are likely to experience dissonance when confronted with information that is inconsistent with their beliefs, attitudes, or behavior (Ahluwalia, 2000). Hence, when trying to maintain the status quo, people are prone to distorting incoming information such that inconsistent information is dismissed or devalued, and consist information is valued as more important. This finding is consistent with research by Innes (1978) demonstrating that highly dogmatic people, who tend to be motivated by reluctance to change, used distorted information processing (e.g., relative weighting and reducing impact) more often than low dogmatic people.

Proposition 2: When people are reluctant to change, they are likely to use empowerment and biased processing strategies to resist persuasion.

### Concerns of Deception

When resistance is motivated by concerns of deception, we argue that *contesting* strategies will be adopted. These strategies can be defined as strategies that resist a persuasion attempt by contesting the content, source, or persuasive strategy of the message. Individuals who are concerned about deception do not want to take the risk of being misinformed. They are motivated to critically process the persuasive message and search for evidence that the message they receive is untrue, untrustworthy, or deceptive (Darke and Ritchie, 2007; Main et al., 2007). In other words, they are more likely to carefully scrutinize the different elements of the message. Because they are motivated by concerns of deception, they are afraid of being misinformed, and tuned toward message cues confirming that the message cannot be trusted. In the advertising literature, the concept of advertising skepticism refers to individuals who distrust the information provided by advertising, and are more likely to critically process advertisements (Obermiller and Spangenberg, 1998). We argue that any contesting strategy may be used in such critical processing. Individuals who are concerned about being misinformed may focus on the inaccuracy of arguments (i.e., contesting the message), the unreliability of the source (i.e., contesting the source), or contest the persuasive strategy that is used.

The result of this processing is a discounting of the persuasive message so that people need not question the accuracy of their existing belief-system. The validity of their beliefs and attitudes remains intact when the message is rejected, and there is no need to incorporate the inconsistent information into one’s belief system when the message can be disregarded and labeled as untruthful (Darke and Ritchie, 2007). Moreover, when people are concerned about being fooled, persuasion knowledge (Friestad and Wright, 1994) is likely to be activated. People will be focused on the strategies that persuaders use to convince them to change their behavior. Recognizing these strategies and labeling them as manipulative and unfair may function as a strategy to resist the message.

Proposition 3: When concerns of deception are present, people are likely to use contesting strategies to resist persuasion.

### Threats to Freedom

Previous research has revealed that threats to freedom are inherently related to *contesting* strategies, particularly contesting the message (i.e., counter arguing). Contesting a message can function as a means of restoring freedom. Fukada (1986) demonstrated that participants who were warned of the persuasive intent of a message and therefore experienced reactance engaged in more counter arguing than participants who were not warned (cf., Dillard and Shen, 2005). Many studies have observed that people engage in counter arguing when their freedoms are threatened. Threats to freedom have previously also been related to source derogation (i.e., contesting the source). For example, Smith (1977) found that participants who were exposed to a threatening message exerted source derogation on three dimensions: objectiveness, expertness, and trustworthiness. Hence, when exposed to threatening information, people evaluate the source of the message as someone less expert, as less objective, and as less trustworthy. Recently, Boerman et al. (2012) revealed that warning participants of the persuasive intent of product placement affected conceptual and attitudinal persuasion knowledge. Being aware of the persuasive intent often arouses reactance, which affects the activation of persuasion knowledge about the strategy that is applied.

People who feel that exposure to a persuasive message threatens their freedom are particularly motivated to restore their freedom. People tend to respond with anger and irritation upon reactance arousal (Brehm and Brehm, 1981). The motivation to restore freedom often results in attitudes or behaviors countering those advocated by the message. When reactance is induced, people may overcorrect whereby the original attitudes and behavior are valued even more than before (Clee and Wicklund, 1980). Therefore, we argue that restoring threatened freedoms can also be achieved through *empowerment* strategies.

Proposition 4: In response to persuasive messages that are perceived to threaten one’s freedom, people are likely to use both contesting and empowerment strategies to resist persuasion.

People can feel threatened in their freedom to (a) hold particular attitudes and behavior, (b) change their attitudes and behavior, and (c) avoid committing to any position or behavior. The type of freedom that is threatened is expected to predict the type of empowerment strategy that people adopt. First, when people experience a threat to retain a particular attitude or behavior they are likely to use the empowerment strategies attitude bolstering and social validation. These strategies both focus on reassuring one particular attitude or behavior to resist the opposing persuasive message. For example, when people feel threatened in their positive attitude toward abortion by exposure to a message against abortion, they are likely to reinforce their existing attitude by thinking about arguments that support their attitude (i.e., attitude bolstering) or by validating their attitudes by important others (i.e., social validations). These strategies are likely to result in even stronger commitment to one’s beliefs, as suggested by reactance theory, and hence reduce persuasion.

Proposition 5a: In response to persuasive messages that are perceived as threatening the freedom to hold a particular attitude or perform a particular behavior, the empowerment strategies of attitude bolstering and social validation, are more likely to be used than the empowerment strategy of asserting confidence.

Second, when resistance is motivated by a more general threat to the freedom of changing attitudes and behavior or by a threat to the freedom to avoid committing to any position or behavior, the empowerment strategy assertions of confidence is more likely to be used. People’s general self-confidence is enhanced when they assert the self. Hence, when people feel that a persuasive message is a threat to their freedom to change attitudes, such as the freedom to feel, think, and behave how they want, they are less likely to be inclined to assert the self to enhance self-esteem. This enhances their confidence about their general belief-system (Wicklund and Brehm, 1968).

Proposition 5b: In response to persuasive messages that are perceived as threatening the more **general** freedom to change or the freedom to avoid committing to any position or behavior, the empowerment strategy of asserting confidence is more likely to be used than other empowerment strategies of resistance.

## General Discussion

By building on existing theory and research, this article presents a preliminary framework explaining why people use certain resistance strategies. This framework provides an initial step to a better understanding of resistance processes. Moreover, this article is the first to present an extensive overview and classification of strategies that people adopt when motivated to resist persuasion. In our framework, we argue that the motives for resistance (i.e., threat to freedom, reluctance to change, and concerns of deception) predict the type of strategy (i.e., avoidance strategies, contesting strategies, biased processing strategies, or empowerment strategies) that people use to resist persuasion.

First, avoidance strategies are proposed to be related to all the identified resistance motives (e.g., freedom threats, reluctance to change, and concerns of deception) because these strategies are assumed to be used upon the anticipated experience of resistance. Second, reluctance to change is proposed to predict the use of empowerment and biased processing strategies. Third, concerns of deception are hypothesized to relate to the adoption of contesting strategies. Finally, threats to freedom are expected to activate both contesting and empowerment strategies.

The presented framework has implications for various fields related to persuasion research, such as health, political, marketing, and organizational communication. For example, the threat to freedom motivation is hypothesized to be related to health messages in particular because people do not prefer others telling them to quit smoking or exercise more, whereas concerns of deception seem more related to marketing messages because people become more skeptical about the trustworthiness of advertising (Obermiller and Spangenberg, 1998). Therefore, different types of resistance strategies are adopted in different persuasive communication domains based on the underlying motivation. Hence, contesting strategies might be used more in marketing communications settings whereas both contesting and empowerment strategies might often be applied in a health communication setting. More knowledge about the situations in which people adopt particular types of resistance strategies might help senders overcome recipients’ resistance.

In addition, it is important to consider the possibility that individuals may differ in their ability to engage in the resistance strategies that are defined here. These differences may not only occur between individuals, but also between strategies within individuals. An individual may be better in employing strategy A than strategy B, which may lead to a preference for one strategy over another. Future research could strive to develop a complete model of resistance that includes not only resistance strategies and their motives, but also individual abilities and situational factors. In addition, such a model could incorporate more complex patterns of resistance, whereby strategies are combined sequentially in response to a persuasion attempt. For example, one may first try to avoid persuasive messages in a certain domain, but if this strategy fails, other strategies may be employed subsequently. For example, Chaiken et al. (1996) find that people ignore threatening information and devote little resources to it. This strategy, however, is not always feasible, so that other strategies need to be employed. One candidate strategy in this particular case may be motivated skepticism (Ditto and Lopez, 1992; Taber and Lodge, 2006). It seems worthwhile to explore the possibility that such sequential use of strategies is path-dependent, with the choice of a strategy at t + 1 depending on the strategy that was used at time t.

Future research in this area could use this framework when investigating resistance. The propositions of the framework about the links between the underlying resistance motives and the use of resistance strategies must be empirically tested. Doing so first requires the development of measures to capture the different resistance motives. Some useful scales have already been developed for the threat to freedom motive (e.g., Dillard and Shen, 2005), while others have not yet been operationalized. Second, there is a need for a scale that measures the relative use of the defined resistance strategies.

Additional research questions may be derived when combining the factors that affect the activation of resistance motives and the different types of resistance strategies. For example, the framework predicts that highly skeptical people use contesting strategies to resist persuasion induced by concerns of deception more frequently, whereas dogmatic people will more frequently adopt empowerment strategies to resist persuasion induced by reluctance to change.

The framework also offers a guideline for communication practitioners who want to persuade people toward behaviors such as giving up smoking or drinking alcohol, buying a product, or voting for a particular political candidate. Awareness of motives and strategies to resist persuasive messages, may be used to improve persuasive messages (See Fransen et al., 2015). For example, when counter arguing is likely to be adopted, practitioners may create two-sided messages in which counter arguments are already addressed (Allen, 1991), or when a threat to freedom is a motive for resistance, disguised communication strategies in which the persuasive intent is less obvious, such as brand placement or entertainment-education, might be helpful in undermining the experience of resistance. As proposed by Moyer-Gusé (2008), narrative entertainment might overcome selective avoidance because when people identify with a character, they might be more willing to consider inconsistent viewpoints and behaviors as they are experiencing the story through the eyes of the character. Self-affirmation is a strategy that may be useful when trying to overcome defensive processing (i.e., empowerment strategies), which is often induced by reluctance to change or threats to freedom. Self-affirmation can be achieved by focusing on other valued aspects of the self, which are unrelated to the message threat (Sherman and Cohen, 2002). This strategy allows people to feel a sense of integrity, which enables them to respond more openly to counter attitudinal messages and reduce the use of empowerment strategies.

The literature on resistance to persuasion has spawned many insights on the various ways in which people may resist persuasion attempts and on how resistance is influenced by other variables. The present article aims to provide an overarching structure for this research and advances several propositions for future research. The framework is rooted in literatures from diverse disciplines that have examined resistance to persuasion. We hope it inspires researchers to connect the different areas of resistance research that have been conducted.

### Conflict of Interest Statement

The authors declare that the research was conducted in the absence of any commercial or financial relationships that could be construed as a potential conflict of interest.
